# Association between stressful life events and depression, anxiety, and quality of life among urban adolescents and young adults in Latin America

**DOI:** 10.3389/fpsyg.2024.1466378

**Published:** 2024-11-07

**Authors:** Francisco Diez-Canseco, Adriana Carbonel, Antonio Bernabe-Ortiz, Natividad Olivar, Carlos Gómez-Restrepo, Mauricio Toyama, José Miguel Uribe-Restrepo, Luis Ignacio Brusco, Liliana Hidalgo-Padilla, Daniela Ramirez-Meneses, Fernando Luis Carbonetti, Karen Ariza-Salazar, Diliniya Stanislaus Sureshkumar, Catherine Fung, Stefan Priebe

**Affiliations:** ^1^CRONICAS Center of Excellence in Chronic Diseases, Universidad Peruana Cayetano Heredia, Lima, Peru; ^2^Department of Psychiatry and Mental Health, School of Medicine, Universidad de Buenos Aires, Buenos Aires, Argentina; ^3^Department of Psychiatry and Mental Health, Pontificia Universidad Javeriana, Bogotá, Colombia; ^4^Department of Clinical Epidemiology and Biostatistics, Pontificia Universidad Javeriana, Bogotá, Colombia; ^5^Hospital Universitario San Ignacio, Bogotá, Colombia; ^6^Unit for Social and Community Psychiatry, Wolfson Institute of Population Health, Queen Mary University of London, London, United Kingdom; ^7^Unit for Social and Community Psychiatry, East London NHS Foundation Trust, London, United Kingdom

**Keywords:** stressful life events, depression, anxiety, quality of life, youth, Latin America

## Abstract

**Background:**

Latin American youth have a high prevalence of mental health disorders and face major socioeconomic and public safety problems. This study assesses the association between stressful life events (SLEs) and depression, anxiety, and quality of life among adolescents and young adults from deprived neighbourhoods of Latin America.

**Methods:**

The sample consisted of 2,402 participants, between adolescents (15–16 years) and young adults (20–24 years), from Bogotá (Colombia), Buenos Aires (Argentina), and Lima (Peru), assessed in 2021–2022 and recruited in education and community settings and social media. We evaluated the most frequent recent and distant SLEs (occurred in the previous year and more than a year ago, respectively), the relationship between SLEs and severity of depression (PHQ-8), anxiety (GAD-7), and quality of life (MANSA), and we tested for differences by gender and age group.

**Results:**

The most common recent and distant SLEs were related to public safety issues and financial distress. Accidents and school suspensions were more frequent among men, and sexual harassment and bullying among women. Every additional reported recent SLE increased the odds of mild, moderate, and severe depression (18, 17, and 25%, respectively) and anxiety (10, 17, and 21%, respectively) symptoms. Similar trends were found with distant SLEs and depression (8, 9, and 11% for mild, moderate, and severe symptoms, respectively) and anxiety (9, 11, and 12%, respectively). Furthermore, a higher number of recent and distant SLEs were associated with lower quality of life (*β* = −0.05, *p* < 0.001, 95% CI [−0.06, −0.04] and β = −0.04, *p* < 0.001, 95% CI [−0.05, −0.03], respectively). The relationship between mental distress and quality of life of recent SLEs seems stronger than the one from distant SLEs, and recent SLEs may have a higher impact on adolescents’ mental health compared with young adults.

**Conclusion:**

Both recent and distant SLEs are related to mental distress and quality of life. Policies and programmes should aim to enhance public and health safety, as well as improve individual, family, and community protective factors that could mitigate the effect of SLEs on Latin American youth.

## 1 Introduction

Adolescence is a period characterised by biological, neurological, and social changes (Patton et al., 2016; United Nations Children’s Fund, 2021b), and is a critical time for the onset of mental health disorders (Solmi et al., 2022). Globally, it is estimated that 1 in 7 (14%) 10–19-year-olds experience mental health conditions (World Health Organization, 2021a), with anxiety and depression being the most prevalent (up 42.9% of all mental disorders) in this age group (United Nations Children’s Fund, 2021b). Similarly, young adults are more susceptible to experiencing depression and anxiety symptoms than older adults (Panchal et al., 2023). Young people report more frequently feeling anxious and depressed than those aged 40 and above (United Nations Children’s Fund, 2021a).

Latin America has a considerably younger population than other regions, with approximately 24% of its population aged between 10 and 24 years old (Pan American Health Organization, 2018). Unfortunately, many of these young people face many challenges rooted in poverty and public insecurity that threaten their mental health (Gibbons and Poelker, 2017). Data from Latin America and the Caribbean (LAC) suggest that among 10–19-year-olds, 14.7% of girls and 15.3% of boys have a mental disorder (United Nations Children’s Fund, 2021b). The per year losses associated with mental health disorders for children and adolescents aged 0–19 from LAC are estimated to be 30.61 billion USD (United Nations Children’s Fund, 2021b). Moreover, the COVID-19 pandemic may have worsened the mental health of the LAC population due to social isolation, lack of healthcare access, family-related stress, illness, mortality, and uncertainty (Uribe-Restrepo et al., 2022; Zhang S. X. et al., 2022).

Exposure to stress has been associated with mood disorders among young people, especially if there are multiple sources of stress at the same time (Thapar et al., 2012; Askeland et al., 2020). Stressful life events (SLEs) are challenging and stressful experiences that are perceived as a threat and/or generate an adverse change in the person (e.g., the death of a family member or financial hardship) (Rauschenberg et al., 2022). They are environmental stressors that can be cumulative and contribute to the vulnerability of having a mental health disorder (Spinhoven et al., 2011). SLEs have been associated with increased psychological stress (Rauschenberg et al., 2022), lower quality of life (Tang et al., 2022), a higher chance of being diagnosed with depression and anxiety (Asselmann et al., 2016; Miloseva et al., 2017; Vidal Bustamante et al., 2020; Ji et al., 2021), and a high rate of suicidality (Gårdvik et al., 2021). It is important to consider that SLEs that occur during crucial developmental stages, such as adolescence, can lead to an increased susceptibility to mental distress by causing long-lasting changes in the nervous, endocrine, and immune systems (Gårdvik et al., 2021).

Depressive and anxious disorders are more prevalent among females, and they also tend to be exposed to more SLEs compared to males (Davis et al., 1999; Harkness et al., 2010; Santomauro et al., 2021). According to Thapar et al. (2012), the hormonal characteristics of women after puberty could increase their sensitivity to stress, which would suggest that SLEs have a greater impact among young women. Moreover, early adolescents have been found to be more vulnerable to the detrimental effects of SLEs on mental health outcomes due to the high uncertainty and instability that characterises this developmental stage (Mann et al., 2014; Nishikawa et al., 2018).

The evidence in Latin America regarding the relationship between SLEs and young people’s well-being is still limited. Funding opportunities in Latin America are still limited, and research initiatives are lacking at both national and institutional levels, which would explain the lack of studies in the region. The available studies, focused on small samples or limited populations (e.g., students from one university), have not assessed the relationship between SLEs and quality of life and have not compared the impact of recent and distant SLEs (Heredia-Ancona et al., 2011; Usuga Jerez et al., 2021).

Our study aims to address some of those limitations, assessing the association between SLEs and depression, anxiety, and quality of life among adolescents and young adults from South America. The specific objectives are (1) to identify the most frequent recent (within the last year) and distant (more than a year ago) SLEs; (2) to evaluate the impact of the SLEs on the quality of life, severity of depression and anxiety symptoms; (3) to compare the impact of recent versus distant SLEs on the quality of life and severity of depression and anxiety symptoms; and (4) to explore differences, by gender and age group (adolescents vs. young adults), in the relationships between recent and distant SLEs and quality of life, severity of depression and anxiety symptoms.

## 2 Materials and methods

### 2.1 Study design and setting

This cross-sectional study uses data gathered between April 2021 and November 2022 as part of the OLA Programme, a study of mental health in urban young people from Latin America (Priebe et al., 2021). The study was conducted in Bogotá (Colombia), Buenos Aires (Argentina), and Lima (Peru), three of the most populated capital cities in Latin America. Argentina, Colombia, and Peru are all upper-middle-income countries characterised by inequality, public insecurity, and weak health and social protection systems.

We recruited a non-randomised sample from educational and community settings and used a self-report questionnaire. We used a checklist of SLEs (Supplementary Data 1) to assess the number and temporality of SLEs; the Patient Health Questionnaire-8 (PHQ-8) to assess symptoms of depression; the General Anxiety Disorder-7 (GAD-7) to assess symptoms of anxiety; and the Manchester Short Assessment of Quality of Life (MANSA) to assess quality of life. To answer our objectives, we used logistic regressions and subgroup analysis.

### 2.2 Participants and recruitment

Participants were adolescents between 15 and 16 years old and young adults between 20 and 24 years old who lived in deprived areas (i.e., economically, socially or environmentally disadvantaged areas) in Bogotá, Buenos Aires, and Lima. The inclusion criteria were: (a) being 15–16 or 20–24 years old; (b) living in the city’s poorest 50% neighbourhoods or districts, according to the United Nations Development Programme’s Human Development Index (United Nations, n.d.) in Bogotá and Lima, and according to the Unsatisfied Basic Needs Index in Buenos Aires (Santos, 2014); and (c) having the ability to give informed consent or assent. In the case of adolescents, informed consent from a parent or tutor was required. Young people with severe mental illness (e.g., psychosis), cognitive impairment, and illiteracy-due to the use of self-report questionnaires-, were excluded. The inclusion and exclusion criteria were self-reported by participants or assessed by the research assistant during the screening stage.

The recruitment of participants varied across cities, reflecting differences in local regulations, restrictions related to COVID-19, and practical options for each local team (Gomez-Restrepo et al., 2023). Participants were recruited in schools, universities, and community settings located in the neighbourhoods or districts eligible for the study. Non-Governmental Organisations and government employment and education schemes facilitated contact with potential participants. Additionally, we used Facebook and Instagram advertisements to reach potential participants.

### 2.3 Sample

As previously stated, this study uses the baseline measure from a cohort study. The baseline sample size was calculated to identify variables that predict recovery from symptoms of depression and anxiety with 90% power, 5% significance level, and 25% dropout rate. Detailed information about the sample size calculation can be found in a previous publication (Priebe et al., 2021).

Our aim was to include 2040 participants across the three cities at baseline to build a cohort. We aimed to include 340 young people in each city (1,020 in total) who met the criteria for symptoms of depression and/or anxiety (PHQ-8 and/or GAD-7 score ≥ 10). Additionally, since male participation in research is challenging and we did not want to overrepresent females, we ensured that at least one-third of the participants were male at the screening stage.

### 2.4 Procedures

Participants who were interested in participating had to undergo at screening process to ensure they met the eligibility criteria. They also needed to provide informed consent or assent. Afterwards, we invited them to complete a paper or online questionnaire with a research assistant on standby to answer any queries. In total, 19 researchers from the three cities were involved in the recruitment and data collection. Assessments were done individually or in groups, and the questionnaire took anywhere between 30 and 60 min to complete. REDCap (Harris et al., 2019), a data collection and management software, was used to record the survey data. In the case of paper questionnaires, a trained research assistant manually entered the data into REDCap.

### 2.5 Variables and instruments

The questionnaire combined sociodemographic questions (i.e., gender, age, education) and scales. In this study, the following variables and scales were used:

#### 2.5.1 Outcomes

##### 2.5.1.1 Symptoms of depression

Patient Health Questionnaire-8 (PHQ-8; Kroenke et al., 2009) is an 8-item questionnaire that measures the presence and severity of symptoms of depression in the last 2 weeks. Each item is scored on a scale of 0 (no day) to 3 (almost every day or 12+ days), and the total score ranges from 0 to 24. Participants’ symptoms were categorised into four groups according to established cut points: non-significant (0–4 points), mild (5–9), moderate (10–14), and moderately severe and severe (15–24) (Kroenke et al., 2009). The PHQ-8 has shown good psychometric properties in samples from South America (Schantz et al., 2017; Villarreal-Zegarra et al., 2023).

##### 2.5.1.2 Symptoms of anxiety

General Anxiety Disorder-7 (GAD-7; Spitzer et al., 2006) is a 7-item questionnaire that measures the presence and severity of symptoms of anxiety in the last two weeks. Each item is scored on a scale of 0 (no day) to 3 (almost every day or 12+ days), and the total score ranges from 0 to 21. Participants’ symptoms were categorised into four groups according to established cut points: non-significant (0–4 points), mild (5–9), moderate (10–14), and severe (15–21) (Spitzer et al., 2006). Studies in South America have found good psychometric properties of the GAD-7 (Camargo et al., 2021; Porto et al., 2022; Zabala et al., 2022; Villarreal-Zegarra et al., 2023).

##### 2.5.1.3 Quality of life

We used the Manchester Short Assessment of Quality of Life (MANSA; Priebe et al., 1999), a 12-item scale that measures people’s satisfaction with 12 different aspects of their lives. This scale uses a 7-point Likert scale (1 = totally dissatisfied, and 7 = totally satisfied), and mean scores range from 1 to 7. This measure has good reliability and validity among samples from high income countries (Björkman and Svensson, 2005; Eklund and Sandqvist, 2006).

#### 2.5.2 Exposures

*Recent and Distant* Stressful Life Events (SLEs) (Supplementary Data 1): The SLEs were measured using an adaptation of the Adolescent Appropriate Life Events Scale (Heubeck and O’Sullivan, 1998). It measures the number of SLEs a person has experienced in the last year (“recent SLEs”) and more than a year ago (“distant SLEs”). The scale includes 30 SLEs experienced by respondents or someone close to them. The Recent SLEs and Distant SLEs variables were created by adding up the number of SLEs experienced in the last year and more than a year ago, respectively. Scores of both variables range from 0 to 30.

#### 2.5.3 Moderators

*Gender:* Male, female, other.

*Age group:* Adolescents (aged 15–16) and young adults (aged 20–24).

#### 2.5.4 Confounding variables

Confounders were selected due to prior studies that indicate that these variables were associated with SLEs, anxiety, depression, and quality of life (Roth and Holmes, 1985; Newcomb and Harlow, 1986; Kumar and George, 2013; Esmaeelzadeh et al., 2018; Zilberman et al., 2019; Lee, 2020; Collier Villaume et al., 2021; Ouyang et al., 2021; Alexander et al., 2022; Tang et al., 2022; Zhang J. et al., 2022; Chuang et al., 2023; Dai and Smith, 2023; Xiang et al., 2024). We have made a Directed Acyclic Graph that shows the role of the confounding variables (Supplementary Figure S1).

##### 2.5.4.1 Parent’s highest level of education completed

We used the highest level of formal education that either parent has completed: no formal education, primary education, secondary education, or higher education. This information was gathered from participants.

##### 2.5.4.2 Substance use

We used questions from the Alcohol, Smoking, and Substance Involvement Screening Test (ASSIST; Humeniuk et al., 2010) to assess if participants ever consumed alcohol, tobacco, marihuana, cocaine, amphetamines, inhalants, sedatives, hallucinogens, and opioids. Additionally, this instrument gathered information about how often they consumed each substance in the last 3 months. The World Health Organization endorses the use of ASSIST in Spanish-speaking countries (World Health Organization and Pan American Health Organization, 2011). Four substance use variables were computed: alcohol, tobacco, marihuana, and illicit drug consumption. For the illicit drug category, we used the substance with the highest usage frequency selected from the following: cocaine, amphetamines, inhalants, sedatives, hallucinogens, and opioids. Each variable had 4 levels: never used, not used in the last 3 months, used monthly or less, and used weekly, daily, or almost daily.

##### 2.5.4.3 Social support

We used the Multidimensional Scale of Perceived Social Support (MSPSS; Zimet et al., 1988), a 12-item scale with a 7-point Likert scale (1 = very strongly disagree and 7 = very strongly agree). Mean scores range from 1 to 7. This scale has been previously used in Latin American contexts and has shown good psychometric properties (Navarro-Loli et al., 2019; Oyarzun Gomez and Iriarte Iluffi, 2020).

##### 2.5.4.4 Social capital

The Short Adapted Social Capital Assessment Tool (SASCAT; De Silva et al., 2007) measures cognitive social capital and structural social capital. Cognitive social capital includes four dichotomous questions (0 = no, 1 = yes) about trust, relationship quality, belonging, and safety in the neighbourhood, with a total score that ranges from 0 to 4. Structural social capital is assessed through three dimensions: group membership (10 questions), support from individuals and groups (21 questions), and citizenship activities (2 questions). In each dimension, the participants receive a score between 0 and 2, where 0 means no memberships to groups, no support sources, or no engagement in citizenship activities; 1 means membership to a group, a source of support, and engagement in one citizenship activity; and 2 means belonging to at least two groups, having at least two sources of support, and engagement in two citizenship activities. After adding the score of each dimension, the total score for structural social capital ranges from 0 to 6.

##### 2.5.4.5 Resilience

We used the Connor-Davidson Resilience Scale (CD-RISC 10; Connor and Davidson, 2003), a 10-item scale with a 5-point Likert scale (1 = never, and 5 = always). The total score was the sum of all items and ranged from 0 to 40. Previous articles have found evidence of the scale’s validity and reliability in Latin American samples (Riveros Munévar et al., 2017; Ugarte et al., 2022; Vélez-Botero and Agudelo-Hernández, 2023).

##### 2.5.4.6 Participation in arts activities

We assessed whether participants regularly participated in an artistic activity in the last 30 days. This was a dichotomous variable (0 = no, 1 = yes).

##### 2.5.4.7 Participation in sports and physical activities

We assessed whether participants regularly participated in an artistic activity in the last 30 days. This was a dichotomous variable (0 = no, 1 = yes).

### 2.6 Data analysis

No data imputation methods were used, and each analysis excluded incomplete observations. “I do not know” answers were recoded as missing values.

Descriptive statistics, including frequencies, medians, and interquartile ranges (IQR), were calculated for each variable. Since SLE measures followed a non-normal distribution, the univariate Kruskal Wallis test was used to compare recent and distant SLE levels by categorical groups (e.g., country, gender, and age group). Univariate logistic regressions were performed between gender, age group, and each SLE.

We used crude and adjusted multinomial logistic regressions to explore the relationship between recent and distant SLEs and depression and anxiety symptoms. Odds ratios (OR) and 95% confidence intervals were reported. A multiple linear regression was used to explore the impact of SLEs on quality of life. We assessed the non-multicollinearity of independent variables, the linearity of variables, the normality and independence of residuals, and the homoscedasticity assumptions.

To identify differences by age group and gender in the association between SLEs and the outcomes, we performed the same crude and adjusted multinomial logistic regressions and multiple regressions for each group: adolescents and young adults, and females and males (excluding participants who did not identify as female nor male since they represented about 1% of the sample).

Data were analysed using R, version 4.4.1 (R Core Team, 2023).

### 2.7 Ethics

The study protocol and instruments were approved by the Institutional Review Boards (IRB) of Universidad de Buenos Aires in Argentina (dated October 2nd, 2020), Pontificia Universidad Javeriana Bogotá in Colombia (ref. FM-CIE-1138-20), Universidad Peruana Cayetano Heredia in Peru (ref. Constancia 581-33-20), and Queen Mary University of London in UK (ref. QMERC2020/02).

Informed consent or assent was obtained for each participant before data collection. For participants under 18 years old, parents or guardians provided informed consent. The consent/assent could be provided by digitally signing the consent/assent document, sending a photo or scan of the signed document, by telephone with an audio recording, adding their signature to a REDCap form, or during an in-person meeting. Each participant was assigned an ID to ensure anonymity, and their identifiable information was stored in a secure location. Respondents who completed the survey received vouchers or cash equivalent to $10 US dollars in each country’s currency.

Participants who had high scores on the depression or anxiety symptoms scales (PHQ-8 ≥ 20 and/or GAD-7 score ≥ 15) received a document with information about depression and anxiety and a list of local mental health services. Additionally, researchers implemented risk management protocols to ensure the participants’ safety and well-being.

## 3 Results

**Figure 1:**
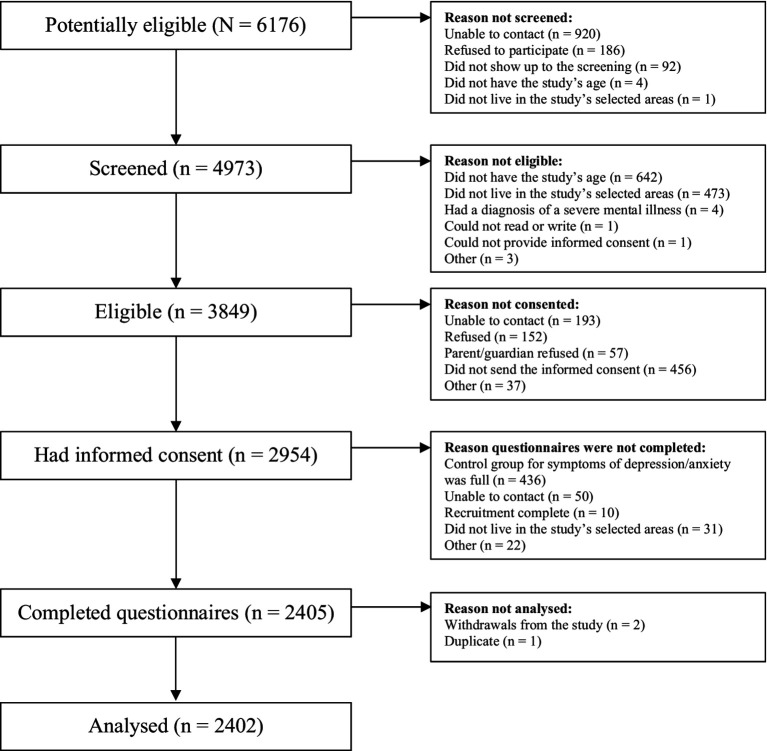
Flowchart of the number of young people at each stage of recruitment.

Figure 1 summarizes the recruitment and screening process. We analysed 2,402 young people across the three cities. As shown in Table 1, most were women (65%), young adults (55%), and students (75%). The median of recent and distant SLEs was 2 (IQR = 1–4) and 7 (IQR = 4–10) events, respectively. 215 (9%) participants had at least one missing value in the variables of interest and covariates.

**Table 1:** Participants’ characteristics and group comparison of recent and distant stressful life events (SLEs).

Variable (complete cases)	*n* (%) [*N* = 2,402]	Recent SLEs, median (IQR)	Recent SLEs statistic*	Distant SLEs, median (IQR)	Distant SLEs statistic*
**Country (*n* = 2,402)**					
Argentina	621 (25.9%)	2 (0–4)	***p* < 0.001**	6 (3–8)	***p* < 0.001**
Colombia	965 (40.2%)	3 (1–5)		6 (4–9)	
Perú	816 (34.0%)	2 (1–4)		8 (5–11)	
**Gender (*n* = 2,399)**					
Male	815 (34.0%)	2 (1–4)	***p* = 0.009**	7 (4–10)	*p* = 0.160
Female	1,560 (65.0%)	2 (1–4)		7 (4–10)	
Other	24 (1.0%)	2.5 (1.75–4.25)		9 (6–10.2)	
**Age group (*n* = 2,402)**					
Adolescents (15–16-year-olds)	1,080 (45.0%)	2 (1–4)	*p* = 0.287	6 (4–9)	***p* < 0.001**
Young adults (20–24-year-olds)	1,322 (55.0%)	2 (1–4)		7 (5–10)	
**Highest level of education completed (*n* = 2,358)**					
None	22 (1.0%)	3 (0.25–5)	*p* = 0.845	6 (4–8.75)	***p* < 0.001**
Primary	1,148 (48.7%)	2 (1–4)		6 (4–9)	
Secondary	1,027 (43.6%)	2 (1–4)		8 (5–10)	
Higher	161 (6.8%)	3 (1–5)		7 (4–11)	
**Main occupation (*n* = 2,402)**					
Work	309 (12.9%)	2 (1–5)	*p* = 0.090	7 (4–10)	*p* = 0.936
Study	1798 (74.9%)	2 (1–4)		7 (4–9)	
Other	295 (12.3%)	2 (1–4)		7 (4–10)	
**Parent’s highest level of education completed (*n* = 2,303)**					
None	149 (6.5%)	3 (1–5)	*p* = 0.100	6 (3–9)	***p* < 0.001**
Primary	492 (21.4%)	2 (1–4)		6 (4–9)	
Secondary	997 (43.3%)	2 (1–4)		7 (4–10)	
Higher	665 (28.9%)	2 (1–4)		7 (5–10)	

*Results from a Kruskal-Wallis test. Bold values denote statistical significance at the p < 0.05 level. IQR, interquartile range.

Regarding mental distress, 23% of the sample did not have significant symptoms of depression, 24% had mild symptoms, 29% moderate symptoms, and 24% severe symptoms, according to their PHQ-8 scores. For anxiety, 25% did not have significant symptoms, 35% had mild symptoms, 27% moderate symptoms, and 14% severe symptoms, according to their GAD-7 scores.

As described in Supplementary Table S1, the most frequent recent SLEs in the whole sample were a close person being mugged or robbed (28%), participants’ household having financial problems (27%), a family member being mugged or robbed (25%), a family member having a life-threatening illness or injury (24%), and a close person’s death (24%). As shown in the Supplementary Table S2, the most frequent distant SLEs were a family member being mugged or robbed (54%), participants’ parents getting a divorce (44%), a close person being mugged or robbed (44%), a close person’s death (42%), and participants being bullied (40%). These tables also reveal important differences in the frequency of some SLEs based on gender. For example, women experienced sexual harassment and bullying much more frequently than men, both in recent and distant time points; men had more accidents anytime; and men were more frequently suspended from school in the past.

The multinomial logistic regressions reveal associations between SLEs and the study outcomes, and these associations were more pronounced for recent SLEs than for distant SLEs (Table 2). The adjusted models show that every additional reported recent SLE increases the odds of having mild, moderate, and severe symptoms of depression by 18, 17, and 25%, respectively. Similarly, it increases the chance of mild, moderate, and severe symptoms of anxiety by 10, 17, and 21%, respectively.

**Table 2:** Multinomial logistic regression for symptoms of depression and anxiety based on recent and distant Stressful Life Events (SLEs).

Outcome	Crude model				Adjusted model^a^			
	Recent SLEs		Distant SLEs		Recent SLEs		Distant SLEs	
	OR	95% CI	OR	95% CI	OR	95% CI	OR	95% CI
**Symptoms of depression**								
Non-significant (ref.)	1		1		1		1	
Mild	1.21***	1.14, 1.27	1.10***	1.06, 1.13	1.18***	1.11, 1.25	1.08***	1.04, 1.12
Moderate	1.24***	1.17, 1.30	1.12***	1.08, 1.15	1.17***	1.10, 1.24	1.09***	1.05, 1.13
Severe	1.33***	1.26, 1.41	1.14***	1.10, 1.18	1.25***	1.17, 1.33	1.11***	1.06, 1.15
**Symptoms of anxiety**								
Non-significant (ref.)	1		1		1		1	
Mild	1.13***	1.08, 1.19	1.11***	1.08, 1.14	1.10***	1.04, 1.15	1.09***	1.05, 1.12
Moderate	1.23***	1.17, 1.29	1.14***	1.10, 1.17	1.17***	1.11, 1.24	1.11***	1.07, 1.15
Severe	1.30***	1.23, 1.37	1.15***	1.11, 1.19	1.21***	1.14, 1.29	1.12***	1.07, 1.17

a Adjusted by gender, age group, parent’s education, substance use, social support, cognitive social capital, structural social capital, resilience, and participation in arts and physical activities. ***p < 0.001.

Furthermore, every additional reported distant SLE increases the odds of having mild, moderate, and severe symptoms of depression by 8, 9, and 11%, respectively. It also increases the risk of mild, moderate, and severe symptoms of anxiety by 9, 11, and 12%, respectively. These estimates from the adjusted model do not change considerably compared with the crude model.

The adjusted multiple linear regression model reveals that a higher number of recent and distant SLEs are associated with a lower quality of life (*β* = −0.05, *p* < 0.001, 95% CI [−0.06, −0.04] and β = −0.04, *p* < 0.001, 95% CI [−0.05, −0.03], respectively). Compared with the crude model, these estimates did not change considerably (Supplementary Table S3). Recent and distant SLEs explain 9% of the variability in quality of life (*R*^2^ = 0.094).

There is not enough statistical evidence to argue that any of the relationships between recent and distant SLEs and the three selected outcomes are influenced by the participant’s gender (Supplementary Tables S4–S7). However, our adjusted analyses show that adolescents have higher odds of experiencing symptoms of depression and anxiety when a recent SLE increases, compared with young adults (Supplementary Tables S8, S9). Specifically, a recent SLE increases the odds of experiencing mild, moderate, and severe depression symptoms by 24, 26, and 39%, respectively, in adolescents, and by 15, 12, and 18% in young adults. Similar patterns and differences between the two age groups are observed with mild, moderate, and severe anxiety symptoms: 12, 22, and 35% in adolescents, and 7, 14, and 14% in young adults.

Moreover, adjusted regressions’ coefficients show a higher inverse association between recent SLEs and quality of life in adolescents compared with young adults (β = −0.08, *p* < 0.001, 95% CI [−0.10, −0.06] and β = −0.03, *p* < 0.001, 95% CI [−0.05, −0.02], respectively) (Supplementary Tables S10, S11). These differences based on respondents’ age group are not as apparent when evaluating the relationship between distant SLEs and the outcome variables (Supplementary Tables S8, S9).

## 4 Discussion

This study aimed to assess the association between stressful life events (SLEs) and depression, anxiety, and quality of life among young people from deprived neighbourhoods in Latin America, identifying the most common SLEs and comparing the impact of recent and distant SLEs by gender and age group.

### 4.1 Most frequent SLEs

The most frequent recent and distant SLEs were related to public safety issues or financial distress, which reflect the context of high delinquency rates and inequality in Latin America and the Caribbean. This region has the highest delinquency rates globally (Machado and Valdés, 2023), and nearly one-third of the region’s population is living in poverty, a percentage that rises to 42.5% in the case of children and adolescents (ECLAC, 2023). Additionally, our sample consisted of young people from deprived neighbourhoods where crime and financial hardship are usually more common.

Other frequent SLEs were the illness or death of family members. Data gathering was during the COVID-19 pandemic when health complications and death of relatives were common globally. South America had the highest rate of children and adolescents who lost their primary or secondary caregivers due to COVID-19 (Hillis et al., 2021). The loss rate was the highest in Peru (14 for every 1,000 children). Therefore, it is likely that this global context influenced the prevalence of death and severe illness of participants’ relatives.

Our results also highlight significant gender disparities in the experience of SLEs. The proportion of young women reporting sexual harassment is between 3.5 (for recent SLEs) to almost 6 times (for distant SLEs) higher than young men. Violence against women affects 1 in 3 women in Latin America and the Caribbean (World Health Organization, 2021b), and Bogotá and Lima are listed as two of the most unsafe capital cities for women in public places (Plan International, 2018). A Colombian study found that 83% of women were harassed while using public transportation at some point in their lives (Veeduría Distrital, 2023). This issue is not as reported in men—specifically heteronormative men—because of the historically sustained gender inequalities that subordinate women.

The findings of this study show that being a victim of bullying had a higher prevalence in women than men. However, the literature results are inconsistent: a study in Latin America also had our finding (Barajas Martínez et al., 2021), but in other regions (Iran and Portugal), men were more likely to be bullied than women (Silva et al., 2013; Mohseny et al., 2019).

Our findings reveal that men had more severe accidents anytime and were more frequently suspended from school in the past. It is well-documented that men have a higher risk for accidents and unintentional injuries than women (Libutzki et al., 2023; World Health Organization, 2023). Since early developmental stages, males are socialised into engaging in more risky behaviour than females and tend to be supervised less by older adults (Udry, 1998; Chou et al., 2022). Additionally, boys are more likely to be suspended or expelled from school than girls (Wallace et al., 2008; Skiba et al., 2014), consistent with our findings.

### 4.2 SLEs impact on mental health

Data show that an additional recent and distant SLE increases the odds of having mild, moderate, and severe symptoms of depression and anxiety. Moreover, more SLEs were associated with a lower quality of life. Previous research has documented the relationship between the number of SLEs and depression and anxiety symptoms (Heredia-Ancona et al., 2011; Asselmann et al., 2016; Barajas Martínez et al., 2021; Ji et al., 2021; Zhao et al., 2023) and quality of life (Pocnet et al., 2016; Tang et al., 2022). SLEs are perceived as a source of threat or harm that requires the individual to adapt (Cohen et al., 2019). Each additional SLE can add to the overall burden of change (Beards et al., 2020; Cohen et al., 2019). Nevertheless, we must consider that some events are recurrent—such as bullying, financial hardship, and sexual harassment—and can lead to chronic stress conditions and associated health risks.

Recent SLEs’ impact on depression and anxiety symptoms and quality of life was stronger than distant SLEs, which has been previously reported (Riese et al., 2014; Nishikawa et al., 2018). Over time, the influence of distant SLEs may diminish as individuals adapt and the events become less central to their current lives (Ormel et al., 2001; Riese et al., 2014). However, exposure to traumatic events during early developmental stages is associated with depressive symptoms, post-traumatic stress symptoms, and other mental health disorders later in life (Phillips et al., 2015; Asselmann et al., 2016; Dunn et al., 2017; Gårdvik et al., 2021). SLEs, caused by people, that are perceived as intentionally harmful, such as physical abuse, neglect, and school violence, tend to be the most traumatic and can lead to long-term mental health issues (Center for Substance Abuse Treatment (US), 2014; Baldwin et al., 2024).

When considering age as a factor that may influence the relationship between SLEs and mental health outcomes, we found that adolescents had higher odds of experiencing symptoms of depression and anxiety when a recent SLE increases compared with young adults. These results are consistent with previous studies (Mann et al., 2014; Nishikawa et al., 2018). Adolescents are finding their identity, are sensitive to social stimuli, and are going through rapid physical and biological changes: the prefrontal cortex, associated with responsible decision-making and impulse control, continues to develop during this stage (Steinberg, 2005; Blakemore, 2012). External stressors may have a higher psychological burden in this age group because these changes provoke uncertainty and instability (Blakemore, 2012; Mann et al., 2014). On the contrary, young adults may have developed better resources to overcome the stress generated by SLEs because of their accumulated life experiences and prefrontal cortex development (Blakemore, 2012; Mann et al., 2014).

Our study did not find gender as a significant moderator of the relationship between SLEs and mental distress outcomes. Although some studies have found that SLEs have a greater impact on depression, anxiety, post-traumatic stress disorder, and neuroticism among women (Mann et al., 2014; Riese et al., 2014), this finding has not been consistently observed in other studies (Davis et al., 1999; Cohen et al., 2019). Further research is needed to explore the role of gender in SLEs and mental health outcomes.

### 4.3 Policy implications

As Cohen et al. (2019) stated, “stressful events do not fall randomly from the sky”; social and environmental circumstances influence them. For instance, compared to high socioeconomic status neighbourhoods, low socioeconomic status neighbourhoods are marked by more frequent and severe stressor exposures (Evans and Kim, 2010; Cohen et al., 2019).

Many of the most common SLEs experienced by adolescents and young adults, such as being a victim of a robbery, suffering a serious illness or injury, going through financial problems, or being a victim of sexual harassment/bullying, are long-standing, multi-causal, and complex problems. These issues are unlikely to be eradicated and require structural changes, as well as strong financial and political commitment. However, there are measures that national and local governments can implement to prevent them and reduce their incidence. For example, governments should focus on crime prevention and prioritise sustainable and safe public spaces where community and leisure activities can occur (Quintero Cordero, 2020). Regarding sexual harassment and bullying, most suffered by women, addressing and raising awareness about these issues early on can lead to long-term positive change. Previous interventions, such as “Benzies and Batchies”, have been effective in preventing sexual harassment in adolescents in different regions, such as Brazil, the Netherlands, and the US (de Lijster et al., 2016; Cruz et al., 2023). Regarding anti-bullying interventions, although rigorous studies in LMICs are still scarce (Sivaraman et al., 2019), the evidence from high-income countries reveals that anti-bullying programmes can be effective at reducing bullying in schools by taking a whole-school approach, supporting students to develop social and emotional competencies, learning ways to respond to bullying behaviours, providing support and training to school staff, and ensuring systematic implementation and evaluation (Centre for Education Statistics and Evaluation, 2023).

The evidence shows that for many mental health disorders, there is time to identify early symptoms and intervene since the first signs can precede a full diagnosis by up to 3 years. Moreover, since the onset of the first symptoms is usually earlier in children who eventually develop a full diagnosis, implementing early measurements could identify those most likely to go on to a full disorder (Costello, 2016). Performing a universal screening in educational settings to identify those youth at risk or currently experiencing mental disorders is a measure for which there is some available evidence. Still, it must be accompanied by the guarantee of care provision and careful protocols to avoid stigmatization (Mental Health America, 2016).

In addition to reducing the occurrence of some SLEs and early detecting young people at higher risk of developing mental disorders, efforts should be made to strengthen protective factors among children, adolescents, and young adults in deprived communities to enhance the management of stress and reduce its detrimental impact on their mental health and quality of life. There are evidence-based interventions to improve, for example, resilience, social support, and social capital (Ordóñez Barba and Ruiz Ochoa, 2015; Dray et al., 2017; García-Carrión et al., 2019; Tasijawa and Siagian, 2022), that could be implemented in schools, universities, communities, and community-based organisations.

Finally, the larger impact of recent SLEs on the well-being of adolescents compared to distant SLEs and young adults could serve to prioritise actions directed at teenagers and give special attention, in classrooms and community organisations, to recent stressful events experienced by this age group.

### 4.4 Strengths and limitations

The study has some strengths to highlight. First, we assessed, with standardised methods, more than 2000 youth participants from underrepresented deprived communities in Latin America. The SLEs’ relationship with mental health has been vastly explored in high-income countries, and in Latin America, studies tend to have small sample sizes. Second, we explored SLEs such as those related to criminality and aggressive events (i.e., bullying, sexual harassment), which may not be as common in high-income countries. Third, we identified which SLEs were the most common in a sample from Latin America, which to our knowledge, has not been previously explored. Fourth, we have provided new insights in the LAC region on the differential impact of recent versus distant SLEs, and comparisons of their impact on the well-being of different age and gender sub-groups.

Our study also has some limitations. Since this was an observational cross-sectional study, we cannot argue causality between SLEs and our outcomes. However, at least regarding the distant SLEs, we know that their occurrence was prior to assessing the mental health outcomes. A second limitation is that we rely on self-reported questionnaires, which can imply memory and social desirability bias. A third limitation is that the high prevalence of depression and anxiety due to the study design and the COVID-19 context may overestimate the association between the variables. Finally, the total SLE scores suggest that each event has the same weight when predicting mental health outcomes, even though we know that some events are usually more severe and can be experienced differently by each person.

## 5 Conclusion

Argentinian, Colombian, and Peruvian youth commonly experience SLEs related to crime, financial hardship, and illness or death of relatives. These, among other SLEs, are associated with an increased likelihood of having depressive and anxiety symptoms and a lower quality of life, especially when the SLEs’ timing was more recent. As a result, policies and social interventions should aim to enhance public and health safety to prevent stressors, as well as improve individual, family, and community protective factors to mitigate the effect of SLEs on Latin American youth. Early intervention should be prioritised since adolescents seem to be more vulnerable than young adults to SLEs.

## Data Availability

The datasets presented will be available beginning 9 months and ending 36 months following article publication. Requests to access the datasets should be directed to Professor Victoria Bird, v.j.bird@qmul.ac.uk.
