# Supplementary material for: Association between stressful life events and depression, anxiety, and quality of life among urban adolescents and young adults in Latin America
Source: Front Psychol. 2024 Nov 7;15:1466378. doi: 10.3389/fpsyg.2024.1466378 (PMC11580160; doi:10.3389/fpsyg.2024.1466378)
Supplement: Supplementary file 1 [file Data_Sheet_1.docx]

Supplementary Material

Association between stressful life events and depression, anxiety, and quality of life among urban adolescents and young adults in Latin America

Francisco Diez-Canseco*, Adriana Carbonel, Antonio Bernabe-Ortiz, Natividad Olivar, Carlos Gómez-Restrepo, Mauricio Toyama, José Miguel Uribe-Restrepo, Luis Ignacio Brusco, Liliana Hidalgo-Padilla, Daniela Ramirez-Meneses, Fernando Luis Carbonetti, Karen Ariza-Salazar, Diliniya Stanislaus Sureshkumar, Catherine Fung, Stefan Priebe

*** Correspondence:** francisco.diez.canseco.m@upch.pe

Supplementary Data 1. Stressful Life Events Checklist

Have any of the following things happened to you during the last 12 months and/or ever in your life?:

|  |  | In the last 12 months | More than a year ago | Never |
| --- | --- | --- | --- | --- |
| 1 | Your parents/carers/siblings/partner/children had a severe illness, injury (e.g., their life was threatened or it had an impact on their daily life) or operation |  |  |  |
| 2 | Someone else very close to you (e.g., a close friend) had a severe illness, injury (e.g., their life was threatened or it had an impact on their daily life) or operation |  |  |  |
| 3 | You had a severe illness, injury (e.g., their life was threatened or it had an impact on their daily life) or operation |  |  |  |
| 4 | You had a serious accident (e.g., an accident requiring hospitalisation) |  |  |  |
| 5 | Your parents/siblings/partner/children had a serious accident (e.g., an accident requiring hospitalisation) |  |  |  |
| 6 | Someone else very close to you (e.g., a close friend) had a serious accident (e.g., an accident requiring hospitalisation) |  |  |  |
| 7 | You moved home |  |  |  |
| 8 | You were temporarily excluded from school |  |  |  |
| 9 | You were permanently excluded from school |  |  |  |
| 10 | You changed primary school |  |  |  |
| 11 | You changed secondary school |  |  |  |
| 12 | You were in care/foster home/children's home |  |  |  |
| 13 | Your family had continuing money problems, e.g., not able to pay rent or bills |  |  |  |
| 14 | Your mum/dad/sibling/partner/child died |  |  |  |
| 15 | Someone else very close to me died (e.g., a close friend) |  |  |  |
| 16 | Your parents divorced or separated |  |  |  |
| 17 | Your parents/carers/partner drank alcohol so often it caused family problems |  |  |  |
| 18 | You were involved in a fire or natural disaster, e.g., flood, earthquake, hurricane |  |  |  |
| 19 | You were a victim of a mugging or robbery |  |  |  |
| 20 | Someone in your family was the victim of a mugging, robbery or burglary |  |  |  |
| 21 | Someone very close to me (e.g., a close friend) was the victim of a mugging, robbery or burglary |  |  |  |
| 22 | You were physically hit and hurt (i.e., assaulted or attacked causing injury) |  |  |  |
| 23 | You were the victim of another crime |  |  |  |
| 24 | You moved here from another region or country or region because your parents/family were fleeing a difficult life (e.g., because of war or violence) |  |  |  |
| 25 | You have been homeless or lived on the street |  |  |  |
| 26 | You cared for your mum, dad, sibling, partner or child due to extended illness or disability |  |  |  |
| 27 | You have been a victim of sexual harassment |  |  |  |
| 28 | You have been a victim of physical or psychological bullying in person or virtually |  |  |  |
| 29 | You lost your job or had to leave your studies |  |  |  |
| 30 | Are there any other difficult experiences that you have lived through?  Specify: ___________________ |  |  |  |

Supplementary Figure 1. Directed Acyclic Graph of stressful life events, depression, anxiety, quality of life, and theoretically and empirically related variables.

Supplementary Table 1. Recent stressful life events’ frequencies and comparisons by gender and age group

| Recent stressful life event | Total, n (%)  [N = 2402] | Gender | | Age-group | |
| --- | --- | --- | --- | --- | --- |
|  |  | Men, n (%)  [N = 815] | Women, n (%)  [N = 1560] | Adolescents (15–16-year-olds), n (%)  [N = 1080] | Young Adults (20–24-year-olds), n (%)  [N = 1322] |
| Someone in your family was the victim of a mugging, robbery or burglary | 1290 (53.7%) | 439 (53.9%) | 837 (53.7%) | 585 (54.2%) | 705 (53.3%) |
| Your parents divorced or separated | 1057 (44.1%) | 340 (41.8%) | 707 (45.4%) | 476 (44.2%) | 581 (44.0%) |
| Someone very close to me (e.g., a close friend) was the victim of a mugging, robbery or burglary | 1048 (43.6%) | 365 (44.8%) | 671 (43.0%) | **441 (40.8%)*** | **607 (45.9%)*** |
| Someone else very close to me died (e.g., a close friend) | 1014 (42.3%) | 342 (42.0%) | 664 (42.6%) | **421 (39.1%)*** | **593 (44.9%)*** |
| You have been a victim of physical or psychological bullying in person or virtually | 957 (39.8%) | **269 (33.0%)*** | **668 (42.8%)*** | 427 (39.5%) | 530 (40.2%) |
| You changed primary school | 910 (37.9%) | 306 (37.5%) | 592 (38.0%) | **438 (40.6%)*** | **472 (35.7%)*** |
| Your family had continuing money problems, e.g., not able to pay rent or bills | 870 (36.2%) | 295 (36.2%) | 561 (36.0%) | **325 (30.1%)*** | **545 (41.2%)*** |
| Your parents/carers/siblings/partner/children had a severe illness, injury (e.g., their life was threatened or it had an impact on their daily life) or operation | 869 (36.2%) | 277 (34.0%) | 580 (37.2%) | 402 (37.2%) | 467 (35.3%) |
| You changed secondary school | 844 (35.1%) | **302 (37.1%)*** | **534 (34.2%)*** | 300 (27.8%) | 544 (41.1%) |
| You moved home | 825 (34.3%) | 286 (35.1%) | 526 (33.7%) | 384 (35.6%) | 441 (33.4%) |
| You were a victim of a mugging or robbery | 821 (34.2%) | **305 (37.4%)*** | **508 (32.6%)*** | **229 (21.2%)*** | **592 (44.8%)*** |
| You have been a victim of sexual harassment | 562 (23.5%) | **73 (9.0%)*** | **472 (30.4%)*** | **207 (19.3%)*** | **355 (26.9%)*** |
| Your parents/carers/partner drank alcohol so often it caused family problems | 557 (23.2%) | 181 (22.2%) | 368 (23.6%) | **227 (21.1%)*** | **330 (25.0%)*** |
| Your parents/siblings/partner/children had a serious accident (e.g., an accident requiring hospitalisation) | 530 (22.1%) | **209 (25.7%)*** | **311 (19.9%)*** | 249 (23.1%) | 281 (21.3%) |
| Someone else very close to you (e.g., a close friend) had a severe illness, injury (e.g., their life was threatened or it had an impact on their daily life) or operation | 515 (21.4%) | 171 (21.0%) | 339 (21.7%) | 240 (22.2%) | 275 (20.8%) |
| You had a severe illness, injury (e.g., their life was threatened or it had an impact on their daily life) or operation | 503 (20.9%) | 174 (21.3%) | 323 (20.7%) | 210 (19.4%) | 293 (22.2%) |
| Someone else very close to you (e.g., a close friend) had a serious accident (e.g., an accident requiring hospitalisation) | 480 (20.0%) | **196 (24.0%)*** | **280 (18.0%)*** | 226 (21.0%) | 254 (19.2%) |
| You were physically hit and hurt (i.e., assaulted or attacked causing injury) | 456 (19.0%) | 166 (20.4%) | 279 (17.9%) | **171 (15.8%)*** | **285 (21.6%)*** |
| You lost your job or had to leave your studies | 399 (16.6%) | 141 (17.3%) | 252 (16.2%) | **24 (2.2%)*** | **375 (28.4%)*** |
| You cared for your mum, dad, sibling, partner or child due to extended illness or disability | 358 (14.9%) | 119 (14.6%) | 233 (14.9%) | 153 (14.2%) | 205 (15.5%) |
| You had a serious accident (e.g., an accident requiring hospitalisation) | 326 (13.6%) | **143 (17.5%)*** | **177 (11.4%)*** | 148 (13.7%) | 178 (13.5%) |
| Are there any other difficult experiences that you have lived through?  Specify: ___________________ | 324 (13.6%) | 95 (11.7%) | 223 (14.4%) | **108 (10.2%)*** | **216 (16.4%)*** |
| You were temporarily excluded from school | 295 (12.3%) | **151 (18.5%)*** | **143 (9.2%)*** | **92 (8.5%)*** | **203 (15.4%)*** |
| Your mum/dad/sibling/partner/child died | 281 (11.7%) | 99 (12.1%) | 179 (11.5%) | **96 (8.9%)*** | **185 (14.0%)*** |
| You were the victim of another crime | 205 (8.5%) | 63 (7.7%) | 137 (8.8%) | **71 (6.6%)*** | **134 (10.1%)*** |
| You were involved in a fire or natural disaster, e.g., flood, earthquake, hurricane | 191 (8.0%) | 68 (8.3%) | 118 (7.6%) | **69 (6.4%)*** | **122 (9.2%)*** |
| You moved here from another region or country or region because your parents/family were fleeing a difficult life (e.g., because of war or violence) | 105 (4.4%) | 33 (4.1%) | 70 (4.5%) | 39 (3.6%) | 66 (5.0%) |
| You were in care/foster home/children's home | 101 (4.2%) | 39 (4.8%) | 60 (3.8%) | 50 (4.6%) | 51 (3.9%) |
| You were permanently excluded from school | 97 (4.0%) | **52 (6.4%)*** | **45 (2.9%)*** | **19 (1.8%)*** | **78 (5.9%)*** |
| You have been homeless or lived on the street | 56 (2.3%) | **26 (3.2%)*** | **29 (1.9%)*** | **8 (0.7%)*** | **48 (3.6%)*** |

* Indicates difference when comparing by gender or age group at *p* < .05. We performed logistic regression analyses to obtain such value.

*Note*: Some gender frequencies may not sum up to the total number since we excluded respondents who did not identify as male or female from this table (n = 24).

Supplementary Table 2. Distant stressful life events’ (SLEs) frequencies and comparisons by gender and age group

| Distant stressful life event | Total, n (%)  [N = 2402] | Gender | | Age-group | |
| --- | --- | --- | --- | --- | --- |
|  |  | Men, n (%)  [N = 815] | Women, n (%)  [N = 1560] | Adolescents (15–16-year-olds), n (%)  [N = 1080] | Young Adults (20–24-year-olds), n (%)  [N = 1322] |
| Someone in your family was the victim of a mugging, robbery or burglary | 1290 (53.7%) | 439 (53.9%) | 837 (53.7%) | 585 (54.2%) | 705 (53.3%) |
| Your parents divorced or separated | 1057 (44.1%) | 340 (41.8%) | 707 (45.4%) | 476 (44.2%) | 581 (44.0%) |
| Someone very close to me (e.g., a close friend) was the victim of a mugging, robbery or burglary | 1048 (43.6%) | 365 (44.8%) | 671 (43.0%) | **441 (40.8%)*** | **607 (45.9%)*** |
| Someone else very close to me died (e.g., a close friend) | 1014 (42.3%) | 342 (42.0%) | 664 (42.6%) | **421 (39.1%)*** | **593 (44.9%)*** |
| You have been a victim of physical or psychological bullying in person or virtually | 957 (39.8%) | **269 (33.0%)*** | **668 (42.8%)*** | 427 (39.5%) | 530 (40.2%) |
| You changed primary school | 910 (37.9%) | 306 (37.5%) | 592 (38.0%) | **438 (40.6%)*** | **472 (35.7%)*** |
| Your family had continuing money problems, e.g., not able to pay rent or bills | 870 (36.2%) | 295 (36.2%) | 561 (36.0%) | **325 (30.1%)*** | **545 (41.2%)*** |
| Your parents/carers/siblings/partner/children had a severe illness, injury (e.g., their life was threatened or it had an impact on their daily life) or operation | 869 (36.2%) | 277 (34.0%) | 580 (37.2%) | 402 (37.2%) | 467 (35.3%) |
| You changed secondary school | 844 (35.1%) | **302 (37.1%)*** | **534 (34.2%)*** | 300 (27.8%) | 544 (41.1%) |
| You moved home | 825 (34.3%) | 286 (35.1%) | 526 (33.7%) | 384 (35.6%) | 441 (33.4%) |
| You were a victim of a mugging or robbery | 821 (34.2%) | **305 (37.4%)*** | **508 (32.6%)*** | **229 (21.2%)*** | **592 (44.8%)*** |
| You have been a victim of sexual harassment | 562 (23.5%) | **73 (9.0%)*** | **472 (30.4%)*** | **207 (19.3%)*** | **355 (26.9%)*** |
| Your parents/carers/partner drank alcohol so often it caused family problems | 557 (23.2%) | 181 (22.2%) | 368 (23.6%) | **227 (21.1%)*** | **330 (25.0%)*** |
| Your parents/siblings/partner/children had a serious accident (e.g., an accident requiring hospitalisation) | 530 (22.1%) | **209 (25.7%)*** | **311 (19.9%)*** | 249 (23.1%) | 281 (21.3%) |
| Someone else very close to you (e.g., a close friend) had a severe illness, injury (e.g., their life was threatened or it had an impact on their daily life) or operation | 515 (21.4%) | 171 (21.0%) | 339 (21.7%) | 240 (22.2%) | 275 (20.8%) |
| You had a severe illness, injury (e.g., their life was threatened or it had an impact on their daily life) or operation | 503 (20.9%) | 174 (21.3%) | 323 (20.7%) | 210 (19.4%) | 293 (22.2%) |
| Someone else very close to you (e.g., a close friend) had a serious accident (e.g., an accident requiring hospitalisation) | 480 (20.0%) | **196 (24.0%)*** | **280 (18.0%)*** | 226 (21.0%) | 254 (19.2%) |
| You were physically hit and hurt (i.e., assaulted or attacked causing injury) | 456 (19.0%) | 166 (20.4%) | 279 (17.9%) | **171 (15.8%)*** | **285 (21.6%)*** |
| You lost your job or had to leave your studies | 399 (16.6%) | 141 (17.3%) | 252 (16.2%) | **24 (2.2%)*** | **375 (28.4%)*** |
| You cared for your mum, dad, sibling, partner or child due to extended illness or disability | 358 (14.9%) | 119 (14.6%) | 233 (14.9%) | 153 (14.2%) | 205 (15.5%) |
| You had a serious accident (e.g., an accident requiring hospitalisation) | 326 (13.6%) | **143 (17.5%)*** | **177 (11.4%)*** | 148 (13.7%) | 178 (13.5%) |
| Are there any other difficult experiences that you have lived through?  Specify: ___________________ | 324 (13.6%) | 95 (11.7%) | 223 (14.4%) | **108 (10.2%)*** | **216 (16.4%)*** |
| You were temporarily excluded from school | 295 (12.3%) | **151 (18.5%)*** | **143 (9.2%)*** | **92 (8.5%)*** | **203 (15.4%)*** |
| Your mum/dad/sibling/partner/child died | 281 (11.7%) | 99 (12.1%) | 179 (11.5%) | **96 (8.9%)*** | **185 (14.0%)*** |
| You were the victim of another crime | 205 (8.5%) | 63 (7.7%) | 137 (8.8%) | **71 (6.6%)*** | **134 (10.1%)*** |
| You were involved in a fire or natural disaster, e.g., flood, earthquake, hurricane | 191 (8.0%) | 68 (8.3%) | 118 (7.6%) | **69 (6.4%)*** | **122 (9.2%)*** |
| You moved here from another region or country or region because your parents/family were fleeing a difficult life (e.g., because of war or violence) | 105 (4.4%) | 33 (4.1%) | 70 (4.5%) | 39 (3.6%) | 66 (5.0%) |
| You were in care/foster home/children's home | 101 (4.2%) | 39 (4.8%) | 60 (3.8%) | 50 (4.6%) | 51 (3.9%) |
| You were permanently excluded from school | 97 (4.0%) | **52 (6.4%)*** | **45 (2.9%)*** | **19 (1.8%)*** | **78 (5.9%)*** |
| You have been homeless or lived on the street | 56 (2.3%) | **26 (3.2%)*** | **29 (1.9%)*** | **8 (0.7%)*** | **48 (3.6%)*** |

* Indicates difference when comparing by gender or age group at *p* < .05. We performed logistic regression analyses to obtain such value.

*Note*: Some gender frequencies may not sum up to the total number since we excluded respondents who did not identify as male or female from this table (n = 24).

Supplementary Table 3. Multiple regression analysis of stressful life events (SLEs) and quality of life

| Variable | Crude model | | | Adjusted model* | | |
| --- | --- | --- | --- | --- | --- | --- |
|  | β | p | 95% CI | β | p | 95% CI |
| Recent SLEs | -.09 | **<.001** | -0.10, -0.07 | -.05 | **<.001** | -0.06, -0.04 |
| Distant SLEs | -.06 | **<.001** | -0.07, -0.05 | -.04 | **<.001** | -0.05, -0.03 |

ᵃAdjusted by gender, age group, parent’s education, substance use, social support, cognitive social capital, structural social capital, resilience, and participation in arts and physical activities.

Supplementary Table 4. Women’s multinomial logistic regression for symptoms of depression and anxiety based on recent and distant stressful life events (SLEs)

| Outcome | Crude model | | | | Adjusted modelᵃ | | | |
| --- | --- | --- | --- | --- | --- | --- | --- | --- |
|  | Recent SLEs | | Distant SLEs | | Recent SLEs | | Distant SLEs | |
|  | OR | 95% CI | OR | 95% CI | OR | 95% CI | OR | 95% CI |
| **Symptoms of depression** |  |  |  |  |  |  |  |  |
| Non-significant (ref.) | 1 |  | 1 |  | 1 |  | 1 |  |
| Mild | 1.24*** | 1.15, 1.34 | 1.07** | 1.02, 1.11 | 1.19*** | 1.10, 1.29 | 1.05 | 1.00, 1.10 |
| Moderate | 1.26*** | 1.18, 1.36 | 1.09*** | 1.04, 1.13 | 1.19*** | 1.10, 1.29 | 1.07** | 1.02, 1.12 |
| Severe | 1.37*** | 1.27, 1.47 | 1.11*** | 1.06, 1.16 | 1.26*** | 1.16, 1.37 | 1.08** | 1.03, 1.14 |
| **Symptoms of anxiety** |  |  |  |  |  |  |  |  |
| Non-significant (ref.) | 1 |  | 1 |  | 1 |  | 1 |  |
| Mild | 1.14*** | 1.06, 1.21 | 1.09*** | 1.04, 1.13 | 1.09* | 1.01, 1.17 | 1.07** | 1.02, 1.12 |
| Moderate | 1.25*** | 1.17, 1.33 | 1.11*** | 1.07, 1.16 | 1.18*** | 1.10, 1.27 | 1.08*** | 1.03, 1.14 |
| Severe | 1.32*** | 1.23, 1.42 | 1.13*** | 1.08, 1.18 | 1.21*** | 1.12, 1.31 | 1.10*** | 1.04, 1.16 |

ᵃ Adjusted by age group, parent’s education, substance use, social support, cognitive social capital, structural social capital, resilience, and participation in arts and physical activities.

* p < .05, ** p < .01, *** p < .001

Supplementary Table 5. Men’s multinomial logistic regression for symptoms of depression and anxiety based on recent and distant stressful life events (SLEs)

| Outcome | Crude model | | | | Adjusted modelᵃ | | | |
| --- | --- | --- | --- | --- | --- | --- | --- | --- |
|  | Recent SLEs | | Distant SLEs | | Recent SLEs | | Distant SLEs | |
|  | OR | 95% CI | OR | 95% CI | OR | 95% CI | OR | 95% CI |
| **Symptoms of depression** |  |  |  |  |  |  |  |  |
| Non-significant (ref.) | 1 |  | 1 |  | 1 |  | 1 |  |
| Mild | 1.17*** | 1.08, 1.27 | 1.13*** | 1.07, 1.18 | 1.18*** | 1.08, 1.29 | 1.13*** | 1.07, 1.19 |
| Moderate | 1.20*** | 1.11, 1.30 | 1.15*** | 1.09, 1.21 | 1.16** | 1.06, 1.27 | 1.11*** | 1.05, 1.18 |
| Severe | 1.29*** | 1.19, 1.41 | 1.18*** | 1.11, 1.24 | 1.26*** | 1.14, 1.40 | 1.14*** | 1.07, 1.22 |
| **Symptoms of anxiety** |  |  |  |  |  |  |  |  |
| Non-significant (ref.) | 1 |  | 1 |  | 1 |  | 1 |  |
| Mild | 1.13*** | 1.05, 1.21 | 1.14*** | 1.09, 1.19 | 1.10* | 1.02, 1.19 | 1.11*** | 1.06, 1.16 |
| Moderate | 1.20*** | 1.11, 1.29 | 1.17*** | 1.12, 1.23 | 1.14** | 1.04, 1.24 | 1.14*** | 1.07, 1.20 |
| Severe | 1.28*** | 1.16, 1.41 | 1.16*** | 1.09, 1.25 | 1.26*** | 1.12, 1.42 | 1.15** | 1.05, 1.26 |

ᵃ Adjusted by age group, parent’s education, substance use, social support, cognitive social capital, structural social capital, resilience, and participation in arts and physical activities.

* p < .05, ** p < .01, *** p < .001

Supplementary Table 6. Women’s multiple regression of recent and distant stressful life events (SLEs) and quality of life

| Variable | Crude model | | | Adjusted modelᵃ | | |
| --- | --- | --- | --- | --- | --- | --- |
|  | β | p | 95% CI | β | p | 95% CI |
| Recent SLEs | -.09 | **<.001** | -0.11, -0.07 | -.05 | **<.001** | -0.07, -0.04 |
| Distant SLEs | -.06 | **<.001** | -0.07, -0.04 | -.04 | **<.001** | -0.05, -0.03 |

ᵃ Adjusted by age group, parent’s education, substance use, social support, cognitive social capital, structural social capital, resilience, and participation in arts and physical activities.

Supplementary Table 7. Men’s multiple regression of recent and distant stressful life events (SLEs) and quality of life

| Variable | Crude model | | | Adjusted modelᵃ | | |
| --- | --- | --- | --- | --- | --- | --- |
|  | β | p | 95% CI | β | p | 95% CI |
| Recent SLEs | -.07 | **<.001** | -0.09, -0.04 | -.05 | **<.001** | -0.07, -0.02 |
| Distant SLEs | -.06 | **<.001** | -0.08, -0.04 | -.04 | **<.001** | -0.05, -0.02 |

ᵃ Adjusted by age group, parent’s education, substance use, social support, cognitive social capital, structural social capital, resilience, and participation in arts and physical activities.

Supplementary Table 8. Adolescents’ multinomial logistic regression for symptoms of depression and anxiety based on recent and distant stressful life events (SLEs)

| Outcome | Crude model | | | | Adjusted modelᵃ | | | |
| --- | --- | --- | --- | --- | --- | --- | --- | --- |
|  | Recent SLEs | | Distant SLEs | | Recent SLEs | | Distant SLEs | |
|  | OR | 95% CI | OR | 95% CI | OR | 95% CI | OR | 95% CI |
| **Symptoms of depression** |  |  |  |  |  |  |  |  |
| Non-significant (ref.) | 1 |  | 1 |  | 1 |  | 1 |  |
| Mild | 1.27*** | 1.16, 1.40 | 1.11*** | 1.05, 1.17 | 1.24*** | 1.12. 1.38 | 1.10** | 1.03, 1.17 |
| Moderate | 1.37*** | 1.25, 1.51 | 1.14*** | 1.08, 1.20 | 1.26*** | 1.13, 1.40 | 1.10** | 1.04, 1.18 |
| Severe | 1.53*** | 1.39, 1.68 | 1.15*** | 1.09, 1.22 | 1.39*** | 1.24, 1.55 | 1.09* | 1.01, 1.17 |
| **Symptoms of anxiety** |  |  |  |  |  |  |  |  |
| Non-significant (ref.) | 1 |  | 1 |  | 1 |  | 1 |  |
| Mild | 1.18*** | 1.09, 1.27 | 1.11*** | 1.06, 1.17 | 1.12* | 1.02, 1.22 | 1.08** | 1.03, 1.14 |
| Moderate | 1.30*** | 1.20, 1.41 | 1.15*** | 1.09, 1.21 | 1.22*** | 1.11, 1.33 | 1.11*** | 1.04, 1.17 |
| Severe | 1.44*** | 1.32, 1.58 | 1.14*** | 1.07, 0.21 | 1.35*** | 1.21, 1.50 | 1.07 | 0.99, 1.15 |

ᵃ Adjusted by gender, parent’s education, substance use, social support, cognitive social capital, structural social capital, resilience, and participation in arts and physical activities.

* p < .05, ** p < .01, *** p < .001

Supplementary Table 9. Young adults’ multinomial logistic regression for symptoms of depression and anxiety based on recent and distant stressful life events (SLEs)

| Outcome | Crude model | | | | Adjusted modelᵃ | | | |
| --- | --- | --- | --- | --- | --- | --- | --- | --- |
|  | Recent SLEs | | Distant SLEs | | Recent SLEs | | Distant SLEs | |
|  | OR | 95% CI | OR | 95% CI | OR | 95% CI | OR | 95% CI |
| **Symptoms of depression** |  |  |  |  |  |  |  |  |
| Non-significant (ref.) | 1 |  | 1 |  | 1 |  | 1 |  |
| Mild | 1.17*** | 1.10, 1.25 | 1.10*** | 1.05, 1.14 | 1.15*** | 1.07, 1.23 | 1.07** | 1.02, 1.12 |
| Moderate | 1.17*** | 1.10, 1.24 | 1.12*** | 1.07, 1.16 | 1.12** | 1.04, 1.20 | 1.08*** | 1.03, 1.13 |
| Severe | 1.23*** | 1.16, 1.32 | 1.15*** | 1.10, 1.20 | 1.18*** | 1.09, 1.27 | 1.11*** | 1.06, 1.17 |
| **Symptoms of anxiety** |  |  |  |  |  |  |  |  |
| Non-significant (ref.) | 1 |  | 1 |  | 1 |  | 1 |  |
| Mild | 1.10** | 1.04, 1.17 | 1.11*** | 1.07, 1.15 | 1.07* | 1.00, 1.15 | 1.09*** | 1.04, 1.14 |
| Moderate | 1.18*** | 1.11, 1.26 | 1.13*** | 1.09, 1.17 | 1.14*** | 1.06, 1.22 | 1.11*** | 1.06, 1.16 |
| Severe | 1.22*** | 1.14, 1.30 | 1.15*** | 1.10, 1.21 | 1.14** | 1.06, 1.24 | 1.14*** | 1.08, 1.21 |

ᵃ Adjusted by gender, parent’s education, substance use, social support, cognitive social capital, structural social capital, resilience, and participation in arts and physical activities.

* p < .05, ** p < .01, *** p < .001

Supplementary Table 10. Adolescents’ multiple regression of recent and distant stressful life events (SLEs) and quality of life

| Variable | Crude model | | | Adjusted modelᵃ | | |
| --- | --- | --- | --- | --- | --- | --- |
|  | β | p | 95% CI | β | p | 95% CI |
| Recent SLEs | -.13 | **<.001** | -0.15, -0.10 | -.08 | **<.001** | -0.10, -0.06 |
| Distant SLEs | -.05 | **<.001** | -0.07, -0.03 | -.03 | **.002** | -0.04, -0.01 |

ᵃ Adjusted by gender, parent’s education, substance use, social support, cognitive social capital, structural social capital, resilience, and participation in arts and physical activities.

Supplementary Table 11. Young adults’ multiple regression of recent and distant stressful life events (SLEs) and quality of life

| Variable | Crude model | | | Adjusted modelᵃ | | |
| --- | --- | --- | --- | --- | --- | --- |
|  | β | p | 95% CI | β | p | 95% CI |
| Recent SLEs | -.06 | **<.001** | -0.08, -0.04 | -.03 | **<.001** | -0.05, -0.02 |
| Distant SLEs | -.06 | **<.001** | -0.07, -0.05 | -.04 | **<.001** | -0.06, -0.03 |

ᵃ Adjusted by gender, parent’s education, substance use, social support, cognitive social capital, structural social capital, resilience, and participation in arts and physical activities.

Supplementary Table 12. Multinomial logistic regression for symptoms of depression and anxiety based on recent and distant stressful life events (SLEs) among participants recruited in educational settings (N = 1129)

| Outcome | Crude model | | | | Adjusted modelᵃ | | | |
| --- | --- | --- | --- | --- | --- | --- | --- | --- |
|  | Recent SLEs | | Distant SLEs | | Recent SLEs | | Distant SLEs | |
|  | OR | 95% CI | OR | 95% CI | OR | 95% CI | OR | 95% CI |
| **Symptoms of depression** |  |  |  |  |  |  |  |  |
| Non-significant (ref.) | 1 |  | 1 |  | 1 |  | 1 |  |
| Mild | 1.22*** | 1.12, 1.33 | 1.13*** | 1.07, 1.19 | 1.20*** | 1.10, 1.32 | 1.14*** | 1.08, 1.21 |
| Moderate | 1.26*** | 1.17, 1.37 | 1.11*** | 1.06, 1.17 | 1.21*** | 1.10, 1.33 | 1.12*** | 1.05, 1.18 |
| Severe | 1.40*** | 1.29, 1.52 | 1.15*** | 1.10, 1.21 | 1.33*** | 1.20, 1.46 | 1.15*** | 1.08, 1.22 |
| **Symptoms of anxiety** |  |  |  |  |  |  |  |  |
| Non-significant (ref.) | 1 |  | 1 |  | 1 |  | 1 |  |
| Mild | 1.19*** | 1.11, 1.28 | 1.10*** | 1.05, 1.15 | 1.14** | 1.05, 1.24 | 1.10*** | 1.04, 1.16 |
| Moderate | 1.29*** | 1.19, 1.39 | 1.14*** | 1.09, 1.20 | 1.24*** | 1.14, 1.36 | 1.15*** | 1.09, 1.22 |
| Severe | 1.36*** | 1.25, 1.48 | 1.16*** | 1.10, 1.22 | 1.27*** | 1.15, 1.40 | 1.13*** | 1.06, 1.21 |

ᵃ Adjusted by age group, parent’s education, substance use, social support, cognitive social capital, structural social capital, resilience, and participation in arts and physical activities.

* p < .05, ** p < .01, *** p < .001

Supplementary Table 13. Multinomial logistic regression for symptoms of depression and anxiety based on recent and distant stressful life events (SLEs) among participants recruited in artistic and non-governmental organizations (N = 333)

| Outcome | Crude model | | | | Adjusted modelᵃ | | | |
| --- | --- | --- | --- | --- | --- | --- | --- | --- |
|  | Recent SLEs | | Distant SLEs | | Recent SLEs | | Distant SLEs | |
|  | OR | 95% CI | OR | 95% CI | OR | 95% CI | OR | 95% CI |
| **Symptoms of depression** |  |  |  |  |  |  |  |  |
| Non-significant (ref.) | 1 |  | 1 |  | 1 |  | 1 |  |
| Mild | 1.31*** | 1.15, 1.48 | 1.06 | 0.98, 1.14 | 1.30*** | 1.12, 1.51 | 1.00 | 0.90, 1.10 |
| Moderate | 1.37*** | 1.20, 1.56 | 1.13** | 1.05, 1.23 | 1.43*** | 1.22, 1.68 | 1.16** | 1.05, 1.30 |
| Severe | 1.33*** | 1.15, 1.54 | 1.18** | 1.07, 1.29 | 1.37** | 1.12, 1.67 | 1.14 | 0.99, 1.31 |
| **Symptoms of anxiety** |  |  |  |  |  |  |  |  |
| Non-significant (ref.) | 1 |  | 1 |  | 1 |  | 1 |  |
| Mild | 1.07 | 0.96, 1.20 | 1.10* | 1.02, 1.19 | 1.07 | 0.93, 1.23 | 1.08 | 0.98, 1.19 |
| Moderate | 1.20** | 1.08, 1.34 | 1.19*** | 1.09, 1.29 | 1.21** | 1.05, 1.40 | 1.17** | 1.05, 1.30 |
| Severe | 1.19* | 1.04, 1.35 | 1.17** | 1.05, 1.30 | 1.17 | 0.98, 1.41 | 1.13 | 0.97, 1.32 |

ᵃ Adjusted by age group, parent’s education, substance use, social support, cognitive social capital, structural social capital, resilience, and participation in arts and physical activities.

* p < .05, ** p < .01, *** p < .001

Supplementary Table 14. Multinomial logistic regression for symptoms of depression and anxiety based on recent and distant stressful life events (SLEs) among participants recruited from governmental programmes (N = 333)

| Outcome | Crude model | | | | Adjusted modelᵃ | | | |
| --- | --- | --- | --- | --- | --- | --- | --- | --- |
|  | Recent SLEs | | Distant SLEs | | Recent SLEs | | Distant SLEs | |
|  | OR | 95% CI | OR | 95% CI | OR | 95% CI | OR | 95% CI |
| **Symptoms of depression** |  |  |  |  |  |  |  |  |
| Non-significant (ref.) | 1 |  | 1 |  | 1 |  | 1 |  |
| Mild | 1.14* | 1.00, 1.30 | 1.03 | 0.95, 1.11 | 1.10 | 0.95, 1.28 | 0.99 | 0.91, 1.09 |
| Moderate | 1.13 | 1.00, 1.29 | 1.05 | 0.97, 1.13 | 1.07 | 0.91, 1.26 | 0.99 | 0.90, 1.09 |
| Severe | 1.31*** | 1.15, 1.49 | 1.05 | 0.97, 1.14 | 1.28** | 1.08, 1.50 | 1.01 | 0.91, 1.12 |
| **Symptoms of anxiety** |  |  |  |  |  |  |  |  |
| Non-significant (ref.) | 1 |  | 1 |  | 1 |  | 1 |  |
| Mild | 1.16* | 1.03, 1.31 | 1.11** | 1.04, 1.20 | 1.16* | 1.01, 1.34 | 1.08 | 0.99, 1.17 |
| Moderate | 1.23** | 1.08, 1.41 | 1.14** | 1.05, 1.24 | 1.24* | 1.05, 1.46 | 1.12* | 1.02, 1.24 |
| Severe | 1.45*** | 1.24, 1.69 | 1.13* | 1.02, 1.26 | 1.45*** | 1.17, 1.80 | 1.21* | 1.03. 1.42 |

ᵃ Adjusted by age group, parent’s education, substance use, social support, cognitive social capital, structural social capital, resilience, and participation in arts and physical activities.

* p < .05, ** p < .01, *** p < .001

Supplementary Table 15. Multinomial logistic regression for symptoms of depression and anxiety based on recent and distant stressful life events (SLEs) among participants who were not recruited from institutions (N = 607)

| Outcome | Crude model | | | | Adjusted modelᵃ | | | |
| --- | --- | --- | --- | --- | --- | --- | --- | --- |
|  | Recent SLEs | | Distant SLEs | | Recent SLEs | | Distant SLEs | |
|  | OR | 95% CI | OR | 95% CI | OR | 95% CI | OR | 95% CI |
| **Symptoms of depression** |  |  |  |  |  |  |  |  |
| Non-significant (ref.) | 1 |  | 1 |  | 1 |  | 1 |  |
| Mild | 1.14* | 1.01, 1.27 | 1.12** | 1.04, 1.21 | 1.10 | 0.97, 1.26 | 1.06 | 0.98, 1.16 |
| Moderate | 1.15* | 1.03, 1.28 | 1.17*** | 1.09, 1.26 | 1.05 | 0.93, 1.19 | 1.08 | 1.00, 1.17 |
| Severe | 1.20** | 1.07, 1.34 | 1.18*** | 1.09, 1.27 | 1.09 | 0.95, 1.24 | 1.07 | 0.98, 1.17 |
| **Symptoms of anxiety** |  |  |  |  |  |  |  |  |
| Non-significant (ref.) | 1 |  | 1 |  | 1 |  | 1 |  |
| Mild | 1.05 | 0.95, 1.16 | 1.11** | 1.04, 1.18 | 1.04 | 0.93, 1.17 | 1.08* | 1.01, 1.16 |
| Moderate | 1.15** | 1.04, 1.27 | 1.11** | 1.04, 1.18 | 1.11 | 0.99, 1.25 | 1.05 | 0.98, 1.14 |
| Severe | 1.21*** | 1.09, 1.35 | 1.15*** | 1.06, 1.24 | 1.17* | 1.02, 1.33 | 1.12* | 1.03, 1.23 |

ᵃ Adjusted by age group, parent’s education, substance use, social support, cognitive social capital, structural social capital, resilience, and participation in arts and physical activities.

* p < .05, ** p < .01, *** p < .001

Supplementary Table 16. Multiple regression of recent and distant stressful life events (SLEs) and quality of life among participants recruited in educational settings (N = 1129)

| Variable | Crude model | | | Adjusted modelᵃ | | |
| --- | --- | --- | --- | --- | --- | --- |
|  | β | p | 95% CI | β | p | 95% CI |
| Recent SLEs | -.09 | **<.001** | -0.11, -0.07 | -.05 | **<.001** | -0.07, -0.03 |
| Distant SLEs | -.05 | **<.001** | -0.07, -0.04 | -.03 | **<.001** | -0.05, -0.02 |

ᵃ Adjusted by age group, parent’s education, substance use, social support, cognitive social capital, structural social capital, resilience, and participation in arts and physical activities.

Supplementary Table 17. Multiple regression of recent and distant stressful life events (SLEs) and quality of life among participants recruited in artistic and non-governmental organizations (N = 333)

| Variable | Crude model | | | Adjusted modelᵃ | | |
| --- | --- | --- | --- | --- | --- | --- |
|  | β | p | 95% CI | β | p | 95% CI |
| Recent SLEs | -.08 | **<.001** | -0.12, -0.04 | -.06 | **.002** | -0.10, -0.02 |
| Distant SLEs | -.06 | **<.001** | -0.09, -0.03 | -.06 | **<.001** | -0.09, -0.02 |

ᵃ Adjusted by age group, parent’s education, substance use, social support, cognitive social capital, structural social capital, resilience, and participation in arts and physical activities.

Supplementary Table 18. Multiple regression of recent and distant stressful life events (SLEs) and quality of life among participants recruited from governmental programmes (N = 333)

| Variable | Crude model | | | Adjusted modelᵃ | | |
| --- | --- | --- | --- | --- | --- | --- |
|  | β | p | 95% CI | β | p | 95% CI |
| Recent SLEs | -.09 | **<.001** | -0.12, -0.05 | -.05 | **.003** | -0.08, -0.02 |
| Distant SLEs | -.03 | **.022** | -0.06, -0.00 | -.01 | .407 | -0.03, -0.01 |

ᵃ Adjusted by age group, parent’s education, substance use, social support, cognitive social capital, structural social capital, resilience, and participation in arts and physical activities.

Supplementary Table 19. Multiple regression of recent and distant stressful life events (SLEs) and quality of life among participants who were not recruited from institutions (N = 607)

| Variable | Crude model | | | Adjusted modelᵃ | | |
| --- | --- | --- | --- | --- | --- | --- |
|  | β | p | 95% CI | β | p | 95% CI |
| Recent SLEs | -.08 | **<.001** | -0.11, -0.05 | -.06 | **<.001** | -0.09, -0.03 |
| Distant SLEs | -.07 | **<.001** | -0.09, -0.05 | -.05 | **<.001** | -0.07, -0.02 |

ᵃ Adjusted by age group, parent’s education, substance use, social support, cognitive social capital, structural social capital, resilience, and participation in arts and physical activities.
